# Solvothermal Synthesis of a Hollow Micro-Sphere LiFePO_4_/C Composite with a Porous Interior Structure as a Cathode Material for Lithium Ion Batteries

**DOI:** 10.3390/nano7110368

**Published:** 2017-11-03

**Authors:** Yang Liu, Jieyu Zhang, Ying Li, Yemin Hu, Wenxian Li, Mingyuan Zhu, Pengfei Hu, Shulei Chou, Guoxiu Wang

**Affiliations:** 1 Laboratory for Microstructures, School of Materials Science and Engineering, Shanghai University, Shanghai 200072, China; yangliu8651@shu.edu.cn (Y.L.); zjy6162@staff.shu.edu.cn (J.Z.); shuliwx@shu.edu.cn (W.L.); zmy@shu.edu.cn (M.Z.); hpf-hqx@shu.edu.cn (P.H.); 2 Shanghai Key Laboratory of Modern Metallurgy & Materials Processing, School of Materials Science and Engineering, Shanghai University, Shanghai 200072, China; 3 Institute for Superconducting and Electronic Materials, University of Wollongong, Wollongong, NSW 2522, Australia; shulei@uow.edu.au; 4 Department of Chemistry and Forensic Science, University of Technology Sydney, Sydney, NSW 2007, Australia; Guoxiu.Wang@uts.edu.au

**Keywords:** lithium ion battery, lithium iron phosphate, solvothermal method, micro hollow sphere

## Abstract

To overcome the low lithium ion diffusion and slow electron transfer, a hollow micro sphere LiFePO_4_/C cathode material with a porous interior structure was synthesized via a solvothermal method by using ethylene glycol (EG) as the solvent medium and cetyltrimethylammonium bromide (CTAB) as the surfactant. In this strategy, the EG solvent inhibits the growth of the crystals and the CTAB surfactant boots the self-assembly of the primary nanoparticles to form hollow spheres. The resultant carbon-coat LiFePO_4_/C hollow micro-spheres have a ~300 nm thick shell/wall consisting of aggregated nanoparticles and a porous interior. When used as materials for lithium-ion batteries, the hollow micro spherical LiFePO_4_/C composite exhibits superior discharge capacity (163 mAh g^−1^ at 0.1 C), good high-rate discharge capacity (118 mAh g^−1^ at 10 C), and fine cycling stability (99.2% after 200 cycles at 0.1 C). The good electrochemical performances are attributed to a high rate of ionic/electronic conduction and the high structural stability arising from the nanosized primary particles and the micro-sized hollow spherical structure.

## 1. Introduction

As one of the most promising polyanion-type cathode materials for high-power Li-ion batteries in electric vehicles (EVs) and energy storage, the olivine-structured LiFePO_4_ has been extensively studied all over the world since 1997. LiFePO_4_ possess numerous appealing features, such as low cost, environmentally benign, good thermal stability, and perfect flat voltage profile at 3.45 V vs. Li^+^/Li [1,2,3]. However LiFePO_4_ suffers from two main disadvantages: low ionic-electronic conductivity (10^−9^–10^−10^ S cm^−1^) and limited lithium ion diffusion channel (one-dimensional path along the b-axis), which significantly restricts the rate performance when attempting fast charging or discharging [4,5]. Tremendous efforts have been exerted to overcome the electronic and ionic transport restriction by optimizing morphology [6,7,8], reducing particle size [9,10,11,12,13], decorating the surface with electricallyconducting agents [14,15,16], and doping the host framework with supervalent cations [17,18,19,20,21]. Among these strategies, size reduction and carbon coating are considered as effective methods to improve the performance of LiFePO_4_, but there still remain some fundamental and technical challenges. Firstly, such electrodes can suffer from undesirable reactions between electrode and electrolyte, which may arise from the high surface area and high surface energies of materials with nano size [9,22]. On the other hand, it is difficult to obtain a full carbon layer coating the surface of LiFePO_4_ particles, so electrons may not reach all the positions where Li^+^ ion intercalation takes place during the charge/discharge process. This can lead to unwanted polarization [14,23,24,25].

Recent research indicates that carbon-coated LiFePO_4_ cathode materials made in the form of hollow microspheres composed of nanoparticles are able to solve the above problems [26,27,28,29,30]. Nanosized particles of LiFePO_4_ can decrease the Li^+^ ion migration lengths and improve the kinetics of LiFePO_4_, while the hollow micro-spherical structure prevents collapse in the long-term cycles and improves the contact between electrode and electrolyte. The most prominent improvement caused by the structure is the small potential drop (or rise) observed just after discharge (or charge) begins at various charge rates. The hydrothermal or solvothermal methods have been demonstrated to be one of most popular routes to fabricate hollow micro-spherical LiFePO_4_ composites with many advantages: mild synthesis conditions, high purity, highly degree of crystallinity, narrow particle size distribution, and vast size ranges. Huang et al. prepared hollow micro-spherical structure LiFePO_4_ with a superior discharge capacity of 101 mAh g^−1^ at 20 C by using carbon spheres as hard templates via the hydrothermal method [31]. However, the hard template methods are time-consuming and costly because of the need for initial synthesis and the final removal of the template. A hollow microspherical LiFePO_4_ material synthesized via hydrothermal method with CTAB as surfactant was reported Lee et al. [32], in which the hollow LiFePO_4_/C composite exhibited a superior energy density of 312 W h g^−1^ at 10 C. However, many impurities were taken into the resultant composite and the balance between morphologies of particles and impurities were studied in another article [33]. Yang et al. [29] also reported monodisperse LiFePO_4_ hollow micro-spheres as high performance cathode materials via solvothermal synthesis using spherical Li_3_PO_4_ as the self-sacrificed template, PEG 600 as a surfactant, and FeCl_2_∙4H_2_O as the Fe^2+^ source in an EG (ethylene glycol) medium. All the above spherical LiFePO_4_ particles were hollow, which results in a waste of the large interior volume, bringing a loss of tap density.

In this work, using inexpensive raw material NH_4_H_2_PO_4_ as P source and CTAB as surfactant in an ethylene glycol (EG) medium, a facile solvothermal route was adopted to synthesize hollow structured LiFePO_4_ spheres with a porous interior which resembles the yolk inside an egg but is composed of carbon-coat primary nanoparticles. The porous spherical shell can permit electrolyte to permeate quickly invade into the interior of the hollow sphere to obtain good contact with the porous “yolk” region, leading to an increasing of specific area, which would facilitate both electronic and lithium ionic diffusion. The as-obtained hollow LiFePO_4_/C microsphere, which was designated as LFP-A, shows a high reversible specific capacity of 163 mAh g^−1^ at 0.1 C, as well as excellent rate capability and cycling performance.

## 2. Results and Discussion 

### 2.1. Microstructure

**Figure 1:**
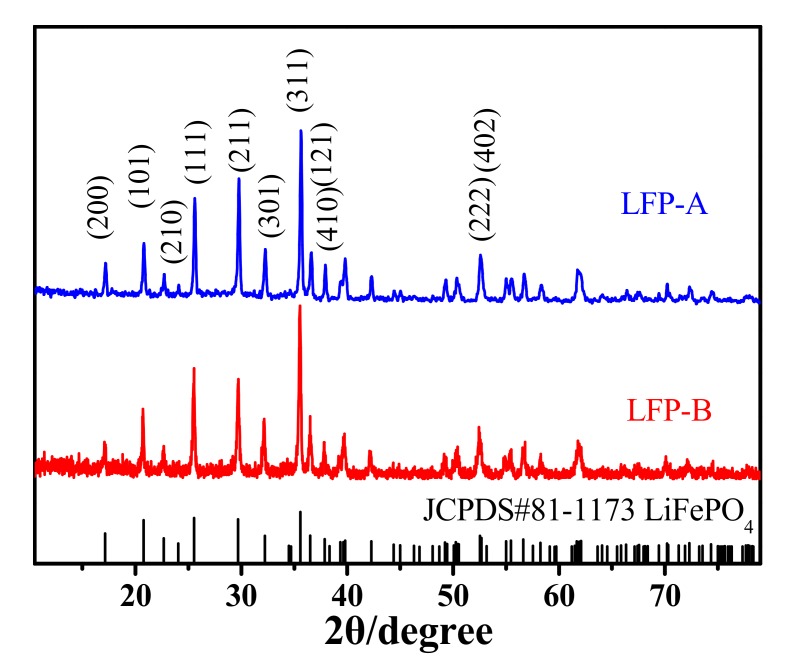
XRD patterns of LFP-A and LFP-B.

The crystal structures of the two composites were investigated by X-ray diffraction. As shown in Figure 1, both samples display the orthorhombic phase with a space group Pmnb (62) (JCPDS card No. 81-1173). The profiles of the reflection peaks are quite narrow, indicating the high crystallinity of the samples. No obvious diffraction peaks of impurity phases (e.g., Fe_2_O_3_, Li_3_PO_4_, and Fe_2_P) are observed in the patterns, which indicate the high purity of the samples. 

**Figure 2:**
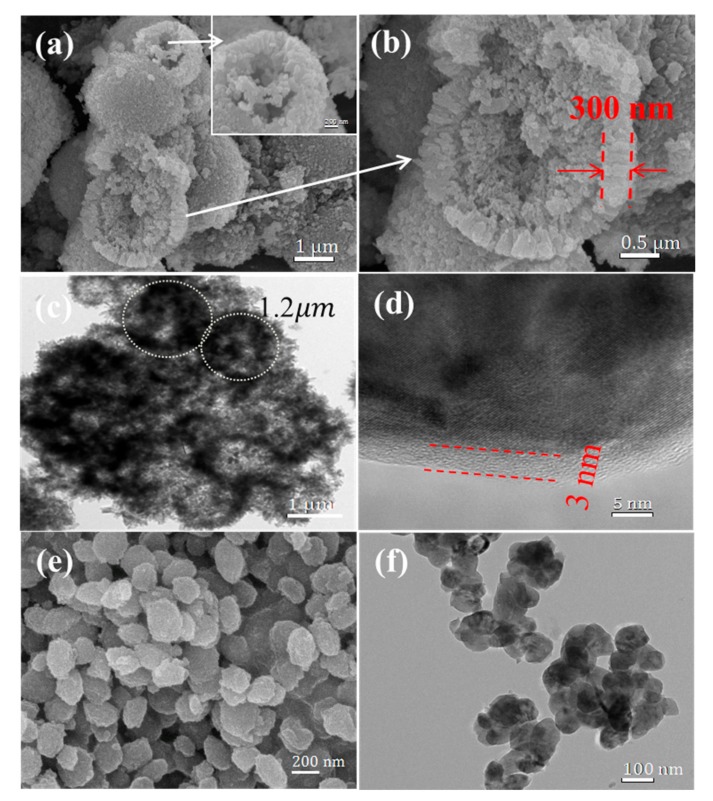
(**a**,**b**) SEM images of LFP-A; (**c**,**d**) TEM images of LFP-A; and (**e**) SEM and (**f**) TEM images of LFP-B.

The scanning electron microscopy (SEM) images of the two samples are shown in Figure 2. Uniform spherical particles with a diameter of approximately 1~3 μm are observed in Figure 2a, and the insert picture shows a broken sphere, exhibiting the likely typical hollow spherical structure of the LFP-A sample. A magnified image of an incomplete microsphere is shown in Figure 2b, indicating that the spherical particles of LFP-A sample is not solid but hollow, with an interior core similar to an egg yolk. It was also observed that the ~300 nm thick porous wall and yolk-interior of the hollow spheres are both composed of ~100 nm primary particles and the yolk is not solid, but porous. The transmission electron microscopy (TEM) micrograph shown in Figure 2c displays a general image of the hollow microspheres of LFP-A sample with a diameter of 1.2 μm. The high-resolution transmission electron microscope (HRTEM) image of the primary particles shown in Figure 2d demonstrates that the nanosized particles are well-crystallized and conformably coated by about 2.5 nm of carbon layers. In contrast, the particles of the sample (LFP-B) synthesized without CTAB display a nanosized spindle-like morphology, which can be clearly observed in the SEM (Figure 2e) and TEM images (Figure 2f). Owing to the novel structure, the LFP-A sample obtains a tap density of 1.2 g cm^−3^, significantly higher than the 0.9 g cm^−3^ measured for the nanosized LFP-B sample. The tap density of the LFP-A sample is similar to the value of other microspherical LiFePO_4_ composites with a solid interior reported recently [34,35,36].

**Figure 3:**
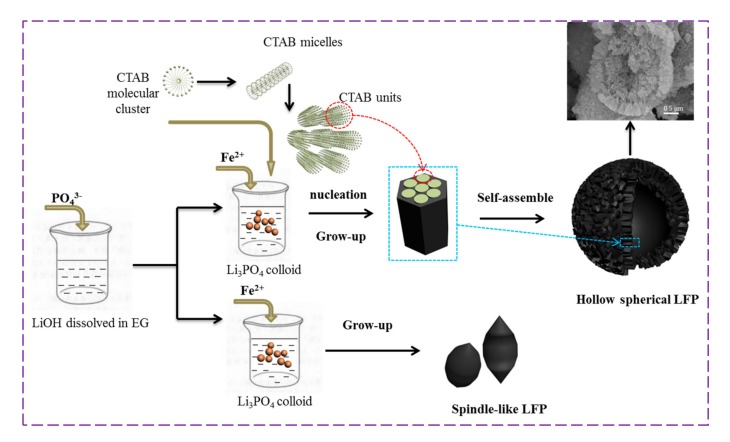
Schematic illustration of the formation of LiFePO_4_/C particles.

The sizes of the primary particles of LFP-A and the whole particles of LFP-B synthesized via our solvothermal route are smaller than that of the particles synthesized by the hydrothermal route in our previous work [21]. The size reduction of particles is attributed to the organic solvent EG which has two hydroxyl groups in its molecule, capable of weak linking to the LFP nanocrystallites via hydrogen bonds, thus inhibiting the growth of the crystals [15]. Comparing the micrographs of the two samples, it can be seen that the morphologies of the powders are completely changed with CTAB involved in the process. The anticipated mechanism for the formation of LiFePO_4_/C particles is shown in Figure 3. An EG-water mixture with a certain fraction of CTAB will self-assemble to form CTAB rod-like micelles in an ordered arrangement. With the decrease of the weight fraction of CTAB as the mixture was introduced into the Li_3_PO_4_ suspension, the growth of CTAB micelles evolves from a hexagonal crystalline phase to an isotropic solution phase [37,38]. Then, hexagonal crystalline micelles will serve as templates in the formation of Fe^2+^-Li_3_PO_4_ composite units which will nucleate further LiFePO_4_ material and the composite units grow in size to form nano-particles. As the reaction proceeds, the particle groups assemble to from spheres owing to the residual CTAB molecules, which gather particles around themselves. After calcination, hollow LiFePO_4_ spheres can be obtained because of the decomposition of CTAB. Therefore, the structure changes, from spindle-like solid particles to hollow microspheres, can be attributed to the presence of the CTAB surfactant. The CTAB in the solvothermal synthesis also works as a carbon source, and is converted to conductive amorphous carbon after heat treatment. Meanwhile, it acts as a carbonaceous-reducing agent during heating to prevent oxidation of Fe^2+^ to Fe^3+^. 

**Figure 4:**
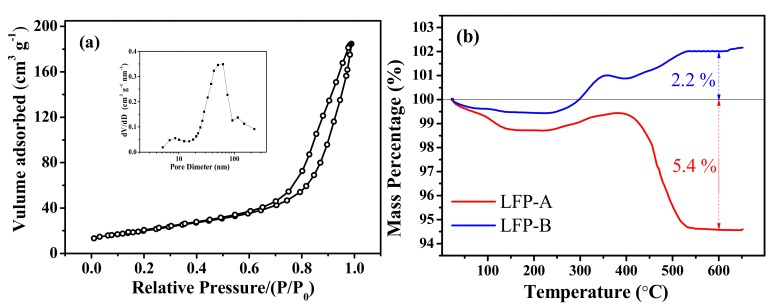
(**a**) Nitrogen adsorption-desorption isotherm curve of LFP-A sample; and (**b**) TG curves of the samples with a heating rate of 10 °C min^−1^ in air.

Figure 4a shows the nitrogen adsorption-desorption isotherms curves for LFP-A sample. The typical-IV curve and H3 hysteresis indicate the presence of mesopores in the as-prepared LiFePO_4_ sample. According to the curve, the specific Brunauer-Emmett-Teller (BET) surface area can be calculated to 31.27 m^2^ g^−1^, and the high specific surface area could provide more sites for lithium ion insertion/extraction, resulting in good electrochemical performance. 

The carbon content in the two samples was estimated by thermogravimetric (TG) measurement in air and the curves are shown in Figure 4b. It should be mentioned that, in the temperature range of 250–500 °C, the olivine LiFePO_4_ can be oxidized to Li_3_Fe_2_(PO_4_)_3_ and Fe_2_O_3_, corresponding to a theoretical weight gain of 5.07%. While the carbon in the samples starts to be oxided to CO_2_ gas above 350 °C, leading to a weight loss, and is burnt out completely above 500 °C, based on the following Equation (1) [39]:

(1) $$\mathrm{LiFeP}{\text{O}}_{4}+1/4{\text{O}}_{2}+x\text{C}+x{\text{O}}_{2}=1/3\text{L}{\text{i}}_{3}\text{F}{\text{e}}_{2}{\left(\text{P}{\text{O}}_{4}\right)}_{3}+1/6\text{F}{\text{e}}_{2}{\text{O}}_{3}+x\text{C}{\text{O}}_{2}$$

where x denotes the carbon content in the composite. By noting the small deviation in mass upon heating, the percentages of carbon in LFP-A and LFP-B are calculated to be 10.77% and 2.87%, respectively. The high carbon content of LFP-A is attributed to the addition of CTAB surfactant and the formation of protective hollow sphere shapes with a porous interior structure. 

### 2.2. Electrochemical Performances

**Figure 5:**
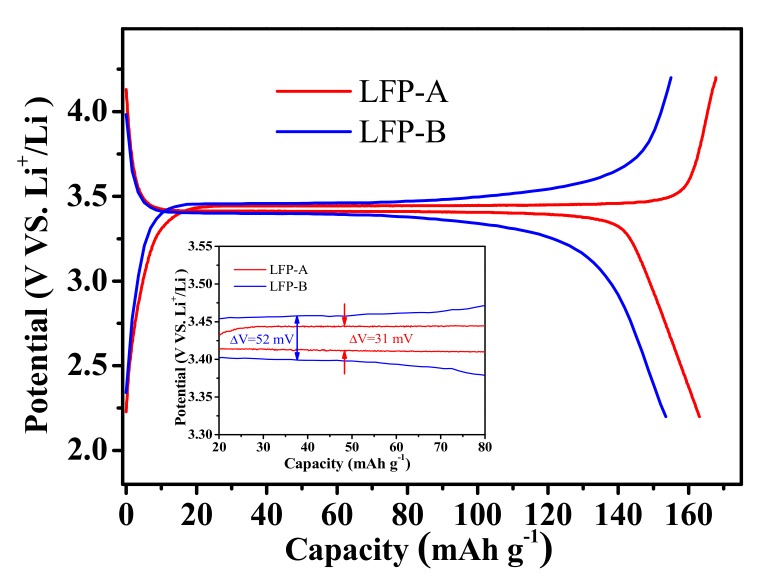
Charge/discharge profiles of the two samples at 0.1 C rate. The inset shows the magnified flat region.

The samples were tested as a cathodic material for lithium ion rechargeable batteries. The loading of electrode material is ca. 1.73 mg cm^−2^, with a 8:1:1 weight ratio of LiFePO_4_/C, acetylene carbon black and poly(vinylidene fluoride). The electrode area is 1.67 cm^2^ with a density of around 1.2 g cm^−3^. The charge/discharge curves of the synthesized LFP samples are shown in Figure 5. LFP-A composite delivers a high specific capacity of 163 mAh g^−1^ at 0.1 C-rate (29.5 × 10^−3^ mA cm^−2^) with a long and flat plateau, which is 95.9% of the theoretical capacity (170 mAh g^−1^). While the LFP-B sample delivers a relatively lower capacity of 153 mAh g^−1^. The LFP-A shows a smaller voltage difference of ca. 31 mV between the charge and discharge plateaus which means it has smaller polarization, i.e., the interfacial electrostatic behavior of the hollow microspherical LiFePO_4_/C composite excel that of the nanosized spindle-like LiFePO_4_/C, which exhibits a voltage difference of ca. 52 mV.

**Figure 6:**
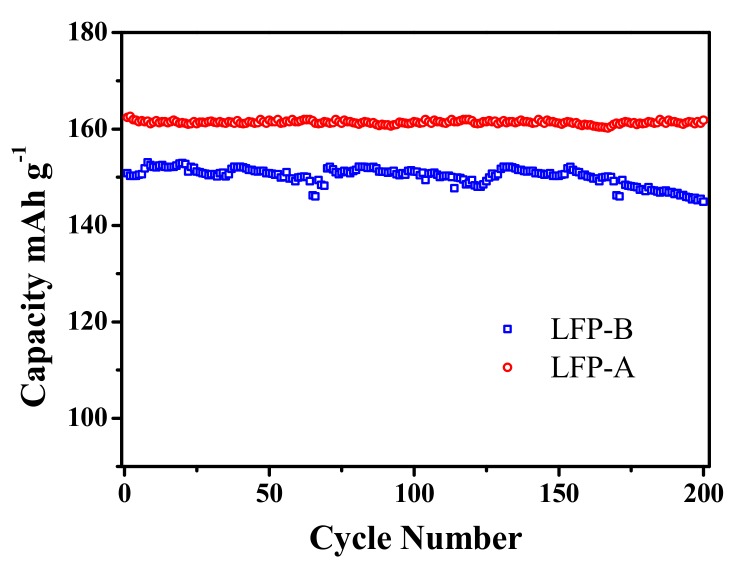
Discharge capacities during continuous cycling of samples at 0.1 C.

The cycling performance of the two samples measured between 2.0 and 4.2 V at a current density of 0.1 C-rate (29.5 × 10^−3^ mA cm^−2^) are displayed in Figure 6. It can be seen that LFP-A and LFP-B samples both exhibit good capacity retention with continuous charge-discharge processes. The capacity of LFP-B sample declines from 152 mAh g^−1^ at the initial cycle to 145 mAh g^−1^ at the 200th cycle, with a capacity loss of 4.5%, while the capacity decay of LFP-A sample after 200 cycles is only 0.8%. The sample composed of hollow microspheres exhibits a better cycling performance, which can be attributed to the microporous hollow spherical structure increasing the contact area between the active materials and the electrolyte. 

**Figure 7:**
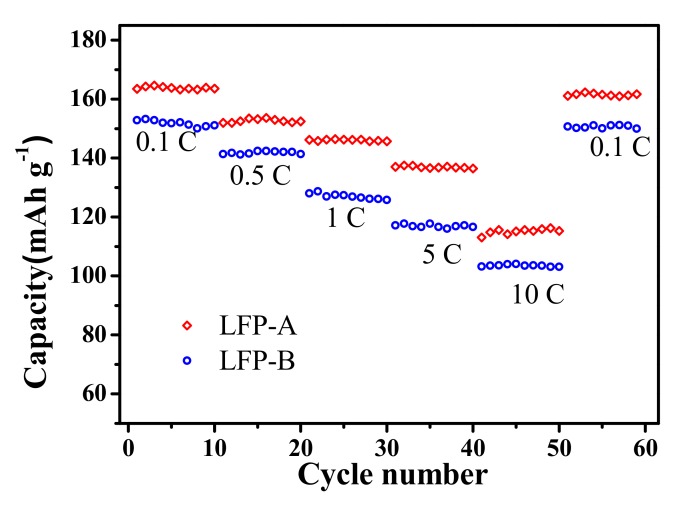
Cyclability of discharge capacities at various charge rates.

Charge and discharge tests at various current rates ranging from 0.1 C to 10 C were performed and the results are shown in Figure 7. The LFP-A sample exhibits a superior rate performance, and capacities as high as 163, 152, 145, 137, and even 118 mA h g^−1^ could be obtained at 0.1, 0.5, 1, 5, and 10 C rates, respectively. The reversible capacity can be recovered and maintained 161.9 mA h g^−1^ when the current rate was again returned to 0.1 C. The corresponding values of the sample synthesized without CTAB are 154, 142, 125, 117, and 102 mA h g^−1^, respectively. The microspherical secondary architecture of hollow LiFePO_4_/C is, hence, suggested to be more favorable for the diffusion of both electrons and ions.

**Figure 8:**
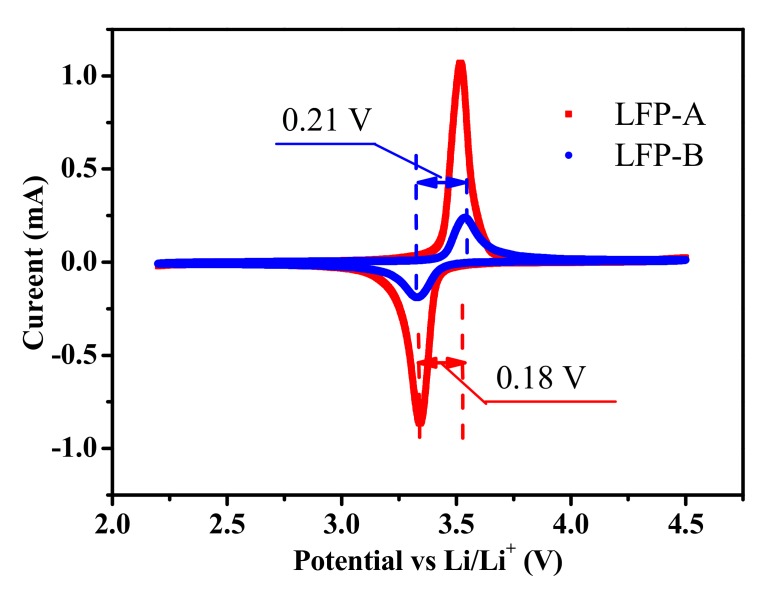
Cyclic voltammograms of the two composites at a scan rate of 0.1 mV s^−1^.

To obtain more information on the electrochemical properties of the two samples, cyclic voltammetry (CV) tests were carried out at a scanning rate of 0.1 mV s^−1^ in the potential range of 2.2–4.5 V. As shown in Figure 8, the LFP-A sample showed a more symmetric shape and sharper peak profiles. It exhibits a pair of anodic and cathodic peaks at 3.52 V and 3.34 V, respectively, resulting in a potential shift of 0.18 V. In the case of the LFP-B composite synthesized without CTAB, the anodic and cathodic peaks were found at 3.54 V and 3.33 V, respectively, with a potential shift of 0.21 V. These peaks correspond to the extraction and insertion of lithium ions and the smaller potential shift illustrates a weaker polarization and possibly an easier kinetic process for LFP-A. It can also be found that the current flow at redox peaks of LFP-A is higher than that in the LFP-B sample. The anodic/cathodic current peaks of LFP-A were about 1.1 mA, while the value for the other sample was about 0.2 mA. These peaks correspond to the extraction and insertion of lithium ions. According to the Randles-Sevcik equation [40], it can be concluded that the Li^+^ ion diffusion in the hollow spherical LiFePO_4_/C is faster than that in the spindle-like LiFePO_4_/C. 

**Figure 9:**
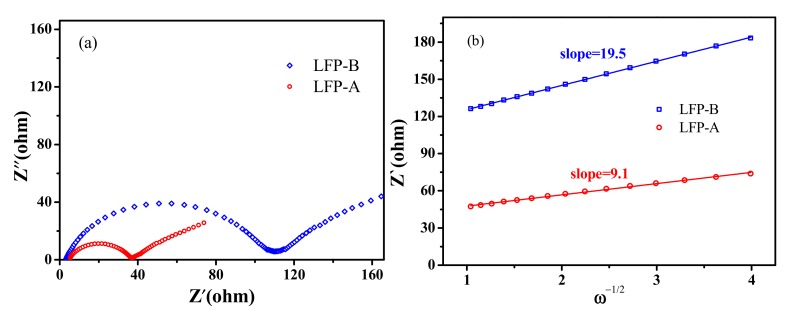
(**a**) Electrochemical impedance spectroscopy (EIS) of the two composites and (**b**) the relationships between Z’ and $\omega^{-1/2}$ in low-frequency regions.

The electrochemical impendance spectra (EIS) plots of the samples are shown in Figure 9a. Both materials exhibit a semicircle in the high-frequency region and a straight line in the low-frequency region. The numerical value of the diameter of the semicircle on the *Z_re_* axis is approximately equal to the charge transfer resistance (*Rct*) [41]. The straight line is attributed to the diffusion of the lithium ions into the bulk of the electrode material, or so-called Warburg diffusion. The lithium-ion diffusion coefficient could be calculated by using the following Equation (2) [42]:

(2) $$D=R^{2}T^{2}/2A^{2}n^{4}F^{4}c^{2}\sigma^{2}$$

where R is the gas constant (8.314 J mol^−1^ K^−1^), T is the absolute temperature, A is the surface area of the cathode, n is the number of electrons per molecule during oxidization, F is the Faraday constant (96,500 C mol^−1^), c is the concentration of lithium ion (7.69 × 10^−3^ mol cm^−3^), and σ is the Warburg factor, which is related with *Z_re_* via Equation (3):

(3) $$Z_{re}=R_{s}+R_{ct}+\sigma \omega^{-1/2}$$

where *Z_re_* represents the real part of the resistance in the low frequency region and ω is the corresponding frequency. The relationships between Z’ and the square root of frequency in the low-frequency region of the two samples are shown in Figure 9b. From the two curves, Li^+^ ion diffusion coefficients (D) of sample LFP-A and LFP-B electrodes are calculated to be 2.32 × 10^−12^ and 5.05 × 10^−13^ cm^2^ s^−1^, respectively. The result indicates that Li ion diffusion in LFP-A is nearly five times faster than that in LFP-B. The values of the diffusion coefficients for the two samples are higher than the literature value for pristine LiFePO_4_ (~10^−14^ cm^2^ s^−1^) [17]. This further confirms that the hollow micro-sphere structure is helpful to the rapid Li ion transport.

Comparing the test results of the two samples, it clearly demonstrates the more excellent electrochemical performance of the LiFePO_4_/C composite with hollow micro-sphere shells and porous interior structure. The distinct improvement in electrochemical performance can be attributed to their successfully designed structural features: (1) the hollow micro-spheres with porous interior structure to prevent structural collapse in long-term cycling by supplying enough space for the change of volume in the extraction and insertion of Li^+^ ions; (2) the nanoparticles comprising the microspheres to promote good electron transfer and enhance the accessibility of lithium ions [9]; and (3) the porous hollow microspherical structure also provides good contact with electrolyte which can flood the interior of the hollow spheres [43]. Even LFP-B, synthesized in the absence of CTAB surfactant, also displays an excellent electrochemical performance, better than that of pristine LiFePO_4_ synthesized via the hydrothermal route in our previous work, and the electrical performance presented in this work is compared with other known hollow spherical LiFePO_4_ composites reported in the literatures, as shown in Table 1. Its high-scoring properties can be attributed to the nano-dimension particle size and the uniform carbon coating layer.

## 3. Materials and Methods 

### 3.1. Synthesis Procedure

All chemicals were analytical grade. The LiFePO_4_/C sample (LFP-A) was prepared by the solvothermal method in an autoclaved stainless steel reactor. The Li_3_PO_4_ white colloids were firstly precipitated from the precursors (LiOH, NH_4_H_2_PO_4_) in EG medium (60% EG + 40% water in volume). Simultaneously, the FeSO_4_ and CTAB were dissolved into EG solution in another container. After 1 h stirring, the mixture solution of FeSO_4_ and CTAB was slowly added into the Li_3_PO_4_ suspension under mild magnetic stirring at room temperature for 15 min. The molar ratio of Li^+^:PO_4_^3−^:Fe^2+^: CTAB was 3:1:1:0.4. Then, the mixture was transferred into a stainless steel autoclave with Teflon lining. The autoclave was sealed and heated at 180 °C for 6 h. The carbon for the LiFePO_4_ coating was sourced from a sucrose solution impregnating-drying-sintering procedure. The sintering was done at 650 °C for 2 h under 5 vol % H_2_/Ar atmosphere with a heating rate of 5 °C min^−1^. As a comparison, another sample named LFP-B was also synthesized via the same process excepting the absence of CTAB surfactant. 

### 3.2. Characterization 

The phase of the sample was analyzed via X-ray diffraction by a Rigaku D\max-2550 X-ray diffractometer (Rigaku, Tokyo, Japan) using Cu K*α*_1_ radiation, and the micro structure and morphology were characterized by field emission scanning electron microscopy (SEM, JEOL JSM6700F, Tokyo, Japan), and high-resolution transmission electron microscopy (HRTEM, JOEL JEM-2010F, Tokyo, Japan). 

### 3.3. Electrochemical Performance 

The electrochemical performances of the samples were evaluated using coin cells (Type 2016). The cathode was prepared by mixing the fabricated powder as active material (80 wt %), acetylene black (10 wt %) and poly(vinylidene fluoride) (PVDF) (10 wt %) dissolved in N-methy1-pyrrolidone (NMP). The slurry was cast onto an Al foil and dried at 80 °C for 12 h. Then the foil was cut into circular discs with a diameter of 1.5 cm by a precision disc cutter (MSK-T06, Hefei KE JING Materials Technology Co., Ltd., HeFei, China). A 1 M LiPF_6_ solution dissolved in a mixture of ethylene carbonate (EC) and dimethyl carbonate (DMC) (EC:DMC = 1:1 in volume) was used as the electrolyte. The cells were assembled in an argon-filled glove box with lithium metal as the anode and microporous polypropylene sheet (Celgard 2400, Celgard, LLC, Charlotte, NC, USA) as a separator.

The cells were tested at different current densities of charge/discharge within the voltage range of 2.5–4.2 V using a LAND battery tester (Wuhan LAND Electronics Co. Ltd., Wuhan, China) at room temperature (25 °C). Cyclic voltammetry (CV) curves were tested at 0.1 mV s^−1^ within the range of 2.2–4.5 V at room temperature by an electrochemical workstation (CHI660E, Shanghai, China). Electrochemical impedance spectroscopy (EIS) was undertaken with an amplitude of 5 mV within the frequency range of 0.01 Hz to 100 kHz by using an Autolab PGSTAT302N (Metrohm, Herisau, Switzerland). 

## 4. Conclusions

In summary, LiFePO_4_/C composite in the form of hollow micro-spheres with a porous interior structure were synthesized through a solvothermal method with the assistance of CTAB surfactant dissolved in an EG solvothermal reaction medium. The addition of CTAB is responsible for the morphology change from spindles to hollow microspheres composed of nanoparticles. The as-obtained LiFePO_4_/C hollow micro-spheres have a small distribution of diameters between 1 and 3 μm, with a ~300 nm thickness porous shell or wall composed of ~100 nm length nanoparticles. The porous shell/wall and loose interior of the hollow spheres make it easy to bring into contact with the electrolyte, facilitating fast, high-volume electronic and lithium ionic diffusion. Electrochemical measurements demonstrated that the hollow spherical LiFePO_4_/C composite displayed excellent electrochemical performances: large reversible discharge capacity of 163 mAh g^−1^ at a current density of 0.1 C-rate (29.5 × 10^−3^ mA cm^−2^), good rate capacity of 103 mAh g^−1^ at the 10 C rate, and superior capacity retention after various current density cycling. The high-scoring results can be attributed to the hollow spherical structure composed of nanosized particles, which facilitates fast electrochemical reaction kinetics and good structural stability. This may provide an effective strategy to produce other LiMPO_4_ (M = Mn, Co, or Ni) hollow micro-spheres as cathode materials for lithium ion batteries. 

## Figures and Tables

**Table 1:** Comparison of hollow spherical LiFePO_4_ composites reported recently.

Surfactant	Size/Primary Particle Size	Initial Capacity (mAh g^−1^, 0.1 C)	Cyclic Performance (mAh g^−1^, 10 C)	Reference
CTAB	1~3 μm/100 nm	163	118	this work
-	200 nm	151	124	[44]
TRITON H-66	30 μm/100–200 nm	-	-	[28]
TRITON H-66	2 μm/100–400 nm	139	96	[27]
-	2 μm	158	101, 20 C, 2000th	[29]
CTAB	240 nm/30–50 nm	135	103	[38]
EDTMP	1–5 μm/20 nm	166	97, 20 C/80, 30 C	[45]
CTAB	420 nm	140	133	[32]
